# Fabrication and Pilot *In Vivo* Study of a Collagen-BDDGE-Elastin Core-Shell Scaffold for Tendon Regeneration

**DOI:** 10.3389/fbioe.2016.00052

**Published:** 2016-06-28

**Authors:** Monica Sandri, Giuseppe Filardo, Elizaveta Kon, Silvia Panseri, Monica Montesi, Michele Iafisco, Elisa Savini, Simone Sprio, Carla Cunha, Gianluca Giavaresi, Francesca Veronesi, Milena Fini, Luca Salvatore, Alessandro Sannino, Maurilio Marcacci, Anna Tampieri

**Affiliations:** ^1^Institute of Science and Technology for Ceramics, National Research Council, Faenza, Italy; ^2^Biomechanics and Technology Innovation Laboratory, Rizzoli Orthopaedic Institute, II Orthopaedic and Traumatologic Clinic, Bologna, Italy; ^3^Nano-Biotechnology Laboratory, Rizzoli Orthopaedic Institute, II Orthopaedic and Traumatologic Clinic, Bologna, Italy; ^4^i3S – Instituto de Investigação e Inovação em Saúde, Universidade do Porto, Porto, Portugal; ^5^Laboratory of Preclinical and Surgical Studies, Rizzoli Orthopaedic Institute, Bologna, Italy; ^6^Laboratory of Biocompatibility, Technological Innovations and Advanced Therapies, Department RIT Rizzoli-Rizzoli Orthopaedic Institute, Bologna, Italy; ^7^Department of Engineering for Innovation, University of Salento, Lecce, Italy

**Keywords:** biomimetic scaffold, collagen, elastin, ligament, tendon, tissue regeneration

## Abstract

The development of bio-devices for complete regeneration of ligament and tendon tissues is presently one of the biggest challenges in tissue engineering. Such device must simultaneously possess optimal mechanical performance, suitable porous structure, and biocompatible microenvironment. This study proposes a novel collagen-BDDGE-elastin (CBE)-based device for tendon tissue engineering, by the combination of two different modules: (i) a load-bearing, non-porous, “core scaffold” developed by braiding CBE membranes fabricated *via* an evaporative process and (ii) a hollow, highly porous, “shell scaffold” obtained by uniaxial freezing followed by freeze-drying of CBE suspension, designed to function as a physical guide and reservoir of cells to promote the regenerative process. Both core and shell materials demonstrated good cytocompatibility *in vitro*, and notably, the porous shell architecture directed cell alignment and population within the sample. Finally, a prototype of the core module was implanted in a rat tendon lesion model, and histological analysis demonstrated its safety, biocompatibility, and ability to induce tendon regeneration. Overall, our results indicate that such device may have the potential to support and induce *in situ* tendon regeneration.

## Introduction

Tendon disorders are common and responsible for marked disability in athletes as well as in non-sportive active population (Jarvinen et al., 2005). The clinical presentation can be acute or chronic, and the pathological findings can range from peri-tendonitis to full-thickness tendon rupture (Filardo et al., 2010). The most common form of tendon healing is by scar formation, with associated compromised joint biomechanics and debilitating symptoms (Longo et al., 2011). The peculiar hierarchical structure of the tendon, a highly anisotropic tissue, in which collagen fibrils assemble in parallel bundles, is difficult to restore since it is organized as an ensemble of structural units at increasing size levels, responsible for its remarkable biofunctional properties. Moreover, the healing process is slow, often incomplete, and with poor tendon vascularization (Longo et al., 2010).

Research efforts have been carried out for decades to investigate suitable materials to reinforce or substitute damaged tendons, i.e., autografts, allografts, and synthetic prostheses have been tested for tendon augmentation. Autografts have been advocated as ideal substitutes for tendon reconstruction, since autologous tissue is endowed with excellent compatibility and ability to revascularize and remodel when transplanted (Janssen et al., 2011). However, significant donor site morbidity associated with this approach has impelled alternative solutions.

Tissue engineering strategies offer potential alternatives to improve tendon repair with the use of scaffolds, growth factors, cell seeding, or combinations of these approaches (Longo et al., 2012). So far, the use of scaffolds has been the most explored strategy due to the possibility of achieving mechanical augmentation along with improving rate and quality of biological healing. One of the major challenges is to design and fabricate a suitable scaffold that may induce specific responses from local cells and achieve the desirable functionality of the tissue to be replaced. Due to the need for strong mechanical properties and ability of suture retention, synthetic scaffolds manufactured from biocompatible chemical compounds may have the ability to off-load the repair at time 0 (Chen et al., 2009). The most common biomaterial designs are electro-spun synthetic and natural polymer mats, particularly PCL (poly-ε-caprolactone), PLLA (poly-l-lactic acid), and collagen, as well as woven fibrous materials (Matthews et al., 2002; Ladd et al., 2011; Liu et al., 2012; Bosworth et al., 2013). Electrospinning is a remarkably simple and versatile technique suitable to generate nanofibers on a large scale allowing to tailor many aspects of the resulting scaffold. Synthetic constructs can be designed with tensile moduli very close to the level of human tendon, while they are able to promote cell alignment, their density do not allow complete cell penetration. Alternatively, scaffolds with tunable 3D microstructural feature, showing significantly higher levels of cells permeability, can be fabricated by electro-spun of natural polymer or by processing mammalian (human, porcine, bovine, and equine) tissues, such as small intestine submucosa, dermis, pericardium, and tendon. However, due to the typically high porosity required to effectively support cell invasion, these materials are orders of magnitude too soft for tendon regeneration (Butler et al., 2008; Kew et al., 2011; Yang et al., 2013). Therefore, it has now become clear that a truly functional tissue-engineering solution requires more than just considering mechanical or structural criteria.

For those reasons, in the present study, a multifunctional composite scaffold mimicking the mechanically efficient core-shell structures found in nature has been proposed for tendon regeneration applications. Following this approach, we have pursued the development of a collagen-based 3D scaffold exhibiting a “core-shell” architecture, in order to synergistically associate the requirements of strength and bioactivity through the coupling of two components differing in their morphological and mechanical properties. Type I collagen has been chosen as base material for the development of the entire core-shell scaffold because it is the major structural protein in most of the soft tissues, thus possessing the ability to assist tissue remodeling and to be resorbed along time by enzymatic action (Browne et al., 2013). Therefore, Type I collagen has several advantages over synthetic polymers investigated to date and can more efficiently drive the critical events of cell adhesion and spreading, as it spontaneously binds many extracellular matrix (ECM) components, such as fibronectin. A suitable cross-linkage with 1,4-butanediol diglycidyl ether (BDDGE) and the addition of water soluble elastin were also performed to further improve the strength, elasticity, and bio-mimicry to tendon tissue, as well as to control the mechanical properties and degradation kinetics of the developed scaffold in physiological environment (Ushiki, 2002; Daamen et al., 2005, 2008).

The core component, a compact and elastic material responsible for the mechanical efficiency of the entire scaffold, was developed by enlacing non-porous collagen-based strips. The shell component, a highly porous hollow tube exhibiting an aligned and interconnected porous microstructure and suitable cell permeability, was produced by means of a uniaxial-freezing technique (Madaghiele et al., 2008) carried out by manipulating freezing and drying parameters to tailor the collagen porosity and microstructural features. This last component was conceived to better support and guide tendon cell infiltration and migration during the regenerative process and promote the scaffold integration and vascularization. In previous works, porous collagen-based scaffolds have been successfully used as regenerative templates for peripheral nerves, cartilage, and skin (Ahmed et al., 2004; Ahn et al., 2010; Kon et al., 2010; Cao et al., 2013; Cerri et al., 2014). The core-shell prototypes developed in this work were subjected to chemical–physical, morphological, and mechanical characterization, to *in vitro* analysis and to *in vivo* implantation, with the aim to document the safety and feasibility of the implant and to achieve preliminary data on its performance in a preclinical model of tendon implant.

## Materials and Methods

### Collagen-BDDGE-Elastin Gel Preparation

Collagen gel in aqueous acetic buffer solution at pH = 3.5, extracted from equine tendon, was supplied by OPOCRIN Spa (Italy). In order to neutralize the acetic acid residuals present in the gel and to reach the maximum grade of collagen fibers self-assembling, the pH was increased with aqueous solution of NaOH (Sigma-Aldrich) 0.5M from pH 3.5 to 5.5 (isoelectric point of collagen). During the titration process, the collagen assumed the appearance of precipitated large fibrous agglomerates that can be well separated from the solvent by centrifugation. The fibers were then washed three times with distilled water by centrifugation at 500 rpm for 5 min, to obtain a homogeneous white-cream colored gel. In order to extend the stability of the collagen scaffolds in physiological conditions, the gel was cross-linked with 1 wt% of BDDGE (Sigma-Aldrich, 95 wt% in aqueous solution) for 48 h at 20°C. The cross-linked gel was washed with distilled water to eliminate possible unreacted BDDGE residuals by three consecutive cycles of dispersion and centrifugation at 500 rpm for 5 min. The cross-linked collagen gel was added to 10 wt% of water soluble elastin (Sigma-Aldrich, from bovine neck ligament) previously dissolved in 2 ml of distilled water and homogenized by magnetic stirring for 1 h.

### Fabrication of the Core Component of the Scaffold

The core component of the scaffold was prepared from thin membranes obtained with collagen-BDDGE-elastin (CBE) gel. The membranes were developed by tape-casting technique matched with an air-drying process in environmental condition aimed to manufacture CBE-based membranes endowed with different thicknesses (150–400 μm) and suitable mechanical properties. Briefly, the CBE gel was spread on Mylar sheet by a tape-casting process to manufacture a thin and uniform film. Thirteen strips 10 cm long and 4 mm wide were cut from the film. To increase the mechanical properties of the final device, three strips at a time, previously soaked in PBS buffer for 20 min at room temperature, were carefully manually enlaced to obtain a stable narrow braid and then air dried. For the *in vivo* evaluation, tendon prototypes (*n* = 10), 2 cm long and 3 mm wide, were manufactured in order to satisfy the requirements for the selected small-size animal model.

### Fabrication of the Shell Component of the Scaffold

A uniaxial-freezing technique followed by freeze-drying was employed to obtain tubular (hollow) and highly porous scaffold characterized by longitudinally oriented pore distribution, according to a protocol described in the literature (Madaghiele et al., 2008), as shell component of the tendon prototypes.

Briefly, the CBE suspension was properly degassed *via* centrifugation and injected into the cylindrical holes (diameter = 5 mm) of a PTFE plate (thickness = 30 mm), sealed at the bottom opening with copper caps provided with an insulating (PTFE) central mandrel (diameter = 3 mm and thickness = 30 mm). Subsequently, the copper base-caps were rapidly cooled (freezing rate = −2°C/min) from room temperature to the final freezing temperature (−40°C) by placing the mold onto the shelf of a freeze-dryer. After freezing, the collagen constructs underwent lyophilization: the ice phase was thus sublimated under vacuum (<100 mTorr) for 17 h at a temperature of 0°C and the frozen solvent removed from the final scaffold structure.

The uniaxial-freezing technique herein described and the subsequent freeze-drying process allowed the production of a tubular structure with a uniform inner diameter. Moreover, the porous structure of the tube wall is characterized by linearly oriented or “axially aligned” pore channels, which potentially define preferential migration patterns.

### Morphological Investigation

Qualitative analyses of membrane and porous scaffold microstructures were performed using a Stereoscan 360 scanning electron microscope (SEM, Leica, UK). The dried membranes and the freeze-dried porous scaffold were previously fixed on SEM specimen Pin Al-stubs and were made electroconductive before the analysis using a Polaron Range sputter-coater (Denton Vacuum, USA) with an Au target.

### Enzymatic Degradation Tests

*In vitro* enzymatic degradation tests were carried out on CBE-based membranes by using collagenase (from *Clostridium histolyticum*; Sigma-Aldrich). Samples (10 mg) were equilibrated for 2 h in PBS at 37°C and then transferred to 5 ml poly-methyl methacrylate cuvettes for UV-VIS spectrophotometric measurements. A solution of collagenase containing 125 CDU/mg (collagen digestion units/mg) in a 0.1M Tris–HCl (pH = 7.4) buffer was prepared, and 3.5 ml was added to 10 mg of each sample. The degradation kinetic was studied using a UV-VIS spectrophotometer (Lambda 35 UV/VIS Spectrometer, Perkin Elmer Instrument, USA), reading the absorbance at λ = 280 nm, corresponding to the wavelength of absorbed radiation of the aromatic amino acids tyrosine and tryptophan, released during the collagen degradation. At scheduled times, the absorption value was measured and used to calculate the % of digested collagen according to the following equation (Eq. 1):

(1) %of digested collagen(t)=[A280(t)/A280 (finalt)] × 100

where A280 (*t*) corresponds to the absorption revealed at time (*t*) and A280 (final *t*) the absorption revealed at the end of the degradation test. The obtained values of collagen digestion rate were reported as a function of time to obtain the degradation kinetic profile of both samples.

### Membrane and Core Tensile Modulus Evaluation

A preliminary screening to select the most suitable membrane in terms of stability, manageability, and elasticity was performed after soaking in PBS at 37°C for 2 h. The membranes (150 μm, 250 μm, 300 μm, and 400 μm) were stretched with a frequency of 1.6 Hz and inducing a deformation of 10% for 100,000 times in wet condition. The applied stress was set up to achieve specimens’ deformation avoiding their rupture. At the end of the cycles, the residual percentage deformation was evaluated and the most performing membranes selected for deeper evaluations.

Tensile tests on the best CBE membranes and core component shaped in form of narrow braids were performed in simulated body conditions (PBS and 37°C) employing a dynamic mechanical thermal analyzer (DMA Q800 TA Instruments, USA) to accomplish static stress/strain measurements. Each sample’s width and thickness were measured and used as input data. To estimate the elastic behavior and specimens’ elongation, the membranes and the braids, previously soaked in PBS at 37°C for 2 h, were stretched in wet conditions with a gradual displacement ramp until the breakage of the specimen. At the end of the analysis, the tensile modulus and the fracture stress were evaluated.

### Swelling Behavior

Collagen-BDDGE-elastin-based porous scaffold and membrane made of pure collagen, used as reference materials, were weighed, placed in PBS solution, and equilibrated for 1 h at 37°C. At scheduled times, the swollen specimen was removed from the medium, and its weight was measured with a Sartorius analytical balance with an accuracy of 0.1 mg. The percentage of swelling degree was calculated on the basis of Eq. (2);

(2) $$S=\frac{\text{Ww}-\text{Wd}}{\text{Wd}}\times 100$$

where *S* is the swelling ratio (%); Ww is the weight of the wet scaffold; and Wd is the initial weight of the dried scaffold. The analysis was carried out until the weight of the wet specimen reached a stable value. The test was performed in triplicate and mean ± SEM plotted on a graph.

### *In Vitro* Cell Morphology Analysis

Mesenchymal stem cells (MSCs) were isolated from rabbit bone marrow and cultured in α-MEM medium plus 10% FBS and 1% Penicillin–Streptomycin (100 U/ml–100 μg/ml). CBE membrane samples were 5.00 mm × 5.00 mm, and CBE porous scaffolds were 5.00 mm × 5.00 mm × 3.00 mm thick, sterilized by 25 kGy γ-ray radiation prior to use.

Samples were placed 1 per well in a 24-well plate and pre-soaked in culture medium; each sample was seeded by carefully dropping 30 μl of cell suspension (1 × 10^4^ cells) onto its surface and allowing cell attachment for 15 min, before addition of 1.5 ml of cell culture medium to each well. All cell handling procedures were performed in a sterile laminar flow hood, and cell culture incubation performed at 37°C with 5% CO_2_. Cell morphology was analyzed at days 1, 3, and 7 by Phalloidin immunofluorescence staining and SEM. In detail for immunofluorescence, cells were fixed with 4% (w/v) paraformaldehyde for 15 min and permeabilized with 1× PBS with 0.1% (v/v) Triton X-100 for 5 min, and FITC-conjugated Phalloidin (Invitrogen) 1:500 was incubated for 20 min at room temperature. Cells were further incubated with DAPI (Invitrogen) for 5 min. Images were acquired by an Inverted Ti-E fluorescence microscope (Nikon). Two samples per time point were analyzed. Cells seeded onto the scaffolds were also analyzed by SEM to examine their morphology. Cells were fixed in 2.5% glutaraldehyde in 0.1M sodium cacodylate buffer pH 7.4 for 2 h at 4°C, washed in 0.1M sodium cacodylate buffer pH 7.4, and dehydrated in a graded series of ethanol. Dehydrated samples were sputter-coated with gold and mounted on a copper grid to be examined at SEM. One sample per time point was analyzed.

### *In Vivo* Tendon Implantation Study

This study was performed in accordance with the Italian Law and the European Legislation on animal experimentation through Directive 2010/63/UE. The research protocol on animals was approved by the Ethical Committee of Rizzoli Orthopedic Institute and by the Italian Ministry of Health. Thirty adult male Sprague Dawley rats (Charles River, Italia), 250–350 g body weight, were housed under controlled conditions and supplied with standard diets and water *ad libitum*. At time of surgery, general anesthesia was induced with an intramuscular injection of 0.6 ml ketamine (Imalgene 1000 Merial Italia SPA, Italy) and 0.5 ml xylazine (Rompun Bayer AG, Germany). Under sterile conditions, a 2-cm midline longitudinal incision was made over the right hind paw of each rat. The Achilles tendon was separated from the surrounding tissue, and a full-thickness tendon defect was created transversely at a point halfway between the musculo-tendinous junction and the insertion onto the calcaneus, when the ankle was brought to a neutral position.

Animals were divided into three groups according to the treatment applied:

(1) Treated group (*n* = 10): a tendon gap of approximately 0.5 cm was made, the defect was filled with the prototype scaffold previously sterilized by 25 kGy γ-ray radiation, and scaffold-tendon suture was applied (6–0 Ethibond suture, Ethicon) according to the Kessler suture technique (Piskin et al., 2007);
(2) Control group (*n* = 10): 0.5 cm length of 2/3 tendon was removed, and a terminal–terminal suture (6–0 Ethibond suture, Ethicon) was applied to maintain the stumps at the correct distance; and
(3) Sham group (*n* = 10): a simulation of the surgery was performed by isolating the tendon from the surrounding tissues, but no tendon lesion was performed.

The skin was sutured with interrupted 2–0 silk sutures. The limb was not immobilized. Every group was divided into two subgroups of five animals each according to experimental times (8 and 16 weeks). The controlateral tendon was used as healthy control for the normalization of histomorphometric parameters.

Postoperatively, antibiotics and analgesics were administered: 0.6 ml/kg flumequil (Flumexil, FATRO SpA, Italy) and 0.1 ml/kg/day metamizole sodium (Farmolisina, Ceva Vetem SpA, Italy). At the end of each experimental time, the animals were anesthetized and then pharmacologically euthanized with an intravenous injection of 1 ml Tanax (Hoechst AG, Germany). Both tendons were explanted, fixed in 10% buffered formalin, and then processed for histological and histomorphometric investigations. After 24 h in formalin, tendons were dehydrated, paraffin-embedded, and sectioned longitudinally in line with the tendon. Sections of 5 μm were obtained with a microtome (Microm HM340E, Thermo Fisher Scientific Inc., Germany) and stained with hematoxylin and eosin, or Picro-Sirius red solution. For histological and histomorphometric analyses, six regions of interest (ROI: 2584 × 1936 pixels) for each slide to nearly cover the entire surface of the tendon were collected at 10× magnification with an optical microscope (Olympus BX51, Olympus Europa Holding GmbH, Germany) coupled to the AxioVision Rel. 4.6 (Carl Zeiss GmbH, Germany) analysis software. A modified semiquantitative score obtained by the three different scores of Soslowsky, Svensson, and Cook was used (Table 1) (Soslowsky et al., 1996; Cook et al., 2004; Svensson et al., 2005). The worst score reachable was 12. Collagen ratio was calculated by automated measurements of the area of thicker, mature collagen fibers, appearing red-orange (type I collagen), versus thinner collagen fibers, appearing pale green (type III collagen), from Picro-sirius red staining (Majewski et al., 2009).

**Table 1:** **Semiquantitative histomorphometric score modified by those of Svensson, Soslowsky, and Cook**.

Parameter	Grade 0	Grade 1	Grade 2	Grade 3
Fiber structure, Svensson and Soslowsky scores	Normal appearance with collagen straight parallel and packed fibers, tangentially oriented with slight waviness	Mild changes with collagen fibers <25% slightly separated with increased waviness	Moderate changes with collagen fibers ≥25% and ≤50% disorganized, separated, and deteriorated	Marked changes with collagen fibers >50% structurally disorganized, and hyalinized
Cellularity (aspect), Cook scores	Inconspicuous elongated spindle nuclei with no obvious cytoplasm	Increased roundness: nucleus becomes more ovoid to round in shape without conspicuous cytoplasm	Increased roundness and size: the nucleus is round, slightly enlarged, and a small amount of cytoplasm is visible	Nucleus is round and large with abundant cytoplasm and lacuna formation
Vascularity, Svensson scores	Vessels run inconspicuous coursing parallel to the collagen fiber bundles in the septa	Slight increase in vessels including transverse vessels in the tendon tissue	Moderate increase in vessels within the tendon tissue	Markedly increased vascularity with vessels
Cartilage formation	No cartilage formation	Isolated hyaline cartilage nodules	Moderate cartilage formation of 25–50%	Extensive cartilage formation, more than 50% of the field involved

### Statistical Analysis

Statistical analysis was performed using the IBM^®^ SPSS^®^ Statistics v.23 software with data reported at a statistical significance level of *p* < 0.05. After having verified normal distribution (Shapiro–Wilks test), the generalized linear model (GLM) with group (treated, control, and sham) and experimental time (8 and 16 weeks) as fixed effects, followed by adjusted Bonferroni *post hoc* test, was used to compare histomorphometric results (normalized total score and collagen ratio). Swelling behavior results were analyzed by two-way analysis of variance, followed by Bonferroni’s *post hoc* test performed by the GraphPad Prism software (version 6.0, La Jolla, CA, USA), with statistical significance set at *p* ≤ 0.05.

## Results and Discussion

**Figure 1:**
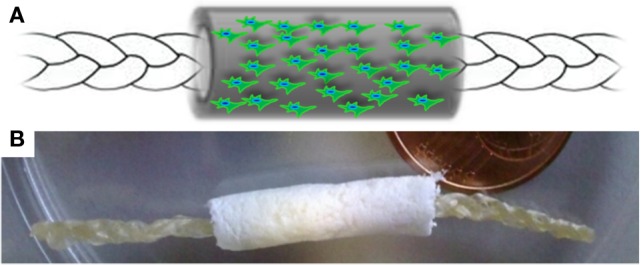
**(A)** Schematic representation and **(B)** macroscopic appearance of the core-shell scaffold architecture. Internal core: high density braided membrane; Outer shell: low density, porous, anisotropic scaffold, with the potential of being seeded by cells. The core was inserted in dry condition into the lumen of the shell (tubular), leaving the extremities of the core module stacked out from the tube, in order to ensure a straightforward suture procedure during surgical operation.

The device proposed in this work was designed to support and stimulate the regeneration of tendon tissues by exploiting the biocompatibility of natural polymers, and the effect of different structural properties of a composite device (i.e., a two-components device). To this aim, a collagen-elastin based biomimetic gel (CBE) was firstly developed, by properly manipulating a raw collagen suspension, and subsequently used to fabricate both components of the “core-shell” device, each of them designed to play specific functional roles. Specifically, the core module of the device, with the appearance of a braided membrane, is devised to work as the load-bearing component of the device, while the shell module, having a tubular (hollow) structure, is meant to act as a physical guide for tendon cells during the regeneration process. The final device is obtained by carefully assembling the two modules (i.e., by simply inserting the core part into the lumen of the shell part), as depicted in Figure 1.

### Collagen-BDDGE-Elastin Gel Preparation

The synthesis consists in a pH-controlled fibration process performed on Type I collagen, allowing to obtain a malleable gel characterized by a collagen supra-molecular assembly. In fact, during the titration process, NaOH (0.5M) solution was used to increase the pH of the collagen gel from 3.5 to 5.5 close to its isoelectric point (pI). At this pH, the collagen molecules were aligned, and the maximum electrostatic and hydrophobic interactions occurred between individual molecules, allowing the self-assembling of the protein quaternary structure and the formation of macroscopic fibrous agglomerates. Due to the induction of the maximum degree of collagen molecular assembling, superior mechanical performances were expected (Silver et al., 2003; Freeman et al., 2005).

Nevertheless, since reconstituted collagen presents typically poor mechanical properties and degrades rapidly *in vivo* due to enzymatic action, a cross-linking reaction by using BDDGE as reticulating agent was performed. BDDGE is a symmetric di-epoxide molecule, and it was selected due to its well-known biocompatibility at low concentration (range 1–3 wt% with respect to collagen) and its ability to react selectively with aminic and/or carboxylic functions of collagen molecules depending on the pH of the reaction environment (Lohre et al., 1992; Zeeman et al., 1999; Tampieri et al., 2008). After a screening of different conditions, the BDDGE solution was added to the collagen gel at pH 5.5 and at a temperature of 25°C in order to reach a good compromise between the molecular assembling and the chemical cross-linking (Nicoletti et al., 2013). The settled condition promotes the cross-linking reaction between the epoxidic groups of BDDGE and the carboxylic groups of collagen, resulting in the formation of ester function linking the two reactive species and producing a flexible and chemically stabilized material. The gel was then enriched with 10 wt% of water soluble elastin (with respect to collagen), since elastin is well known for its ability to promote *in vivo* soft-tissue regeneration and to accelerate synthesis of elastic fibers (Ushiki, 2002; Daamen et al., 2005, 2008). The obtained CBE gel was used to manufacture both the core and the shell components.

### Characterization of the Core Component

**Figure 2:**
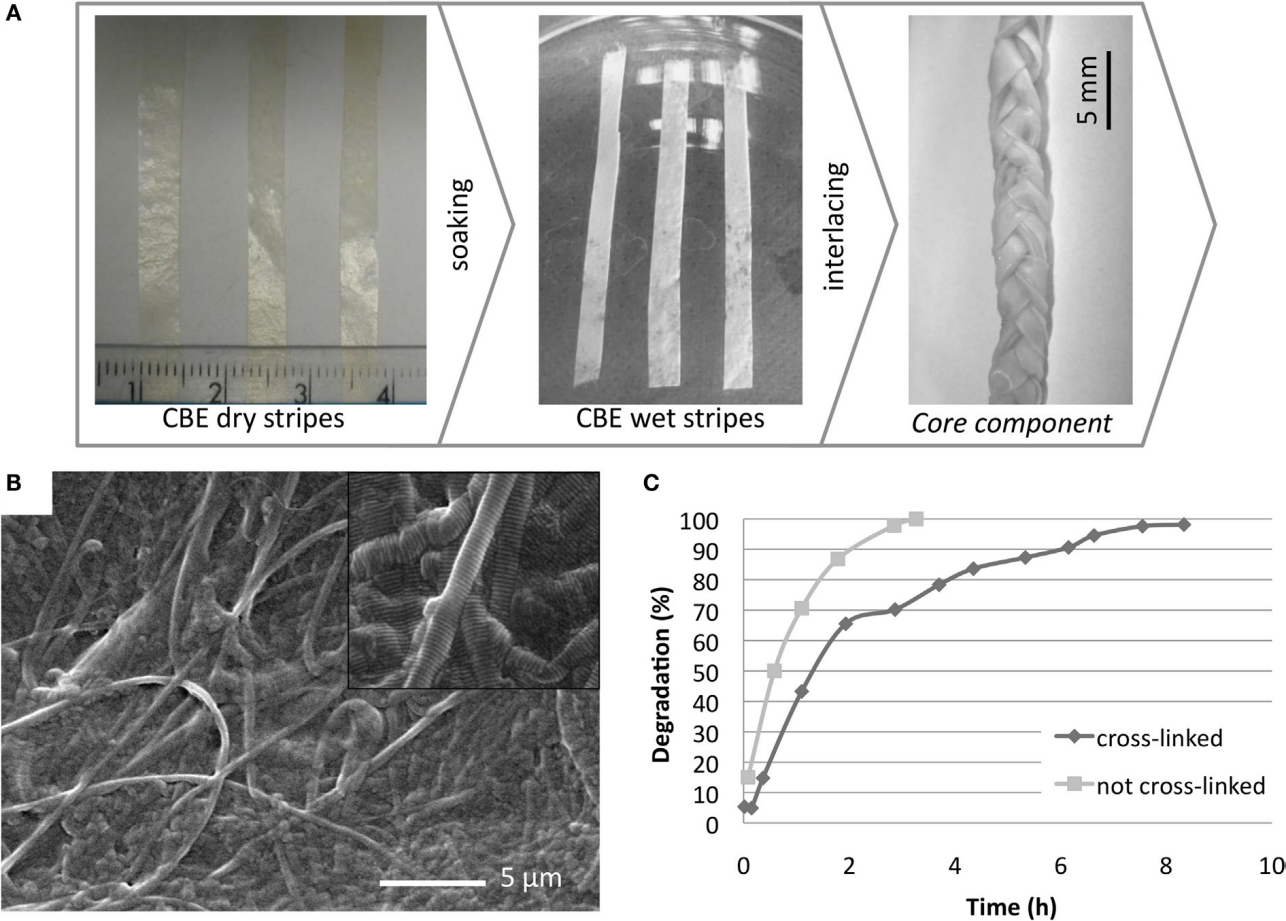
**(A)** Fabrication steps to develop the core component. **(B)** SEM micrograph performed on the CBE membrane surface morphology; **(C)** enzymatic degradation kinetics of CBE membrane cross-linked (◆) and not cross-linked (■) with BDDGE.

**Figure 3:**
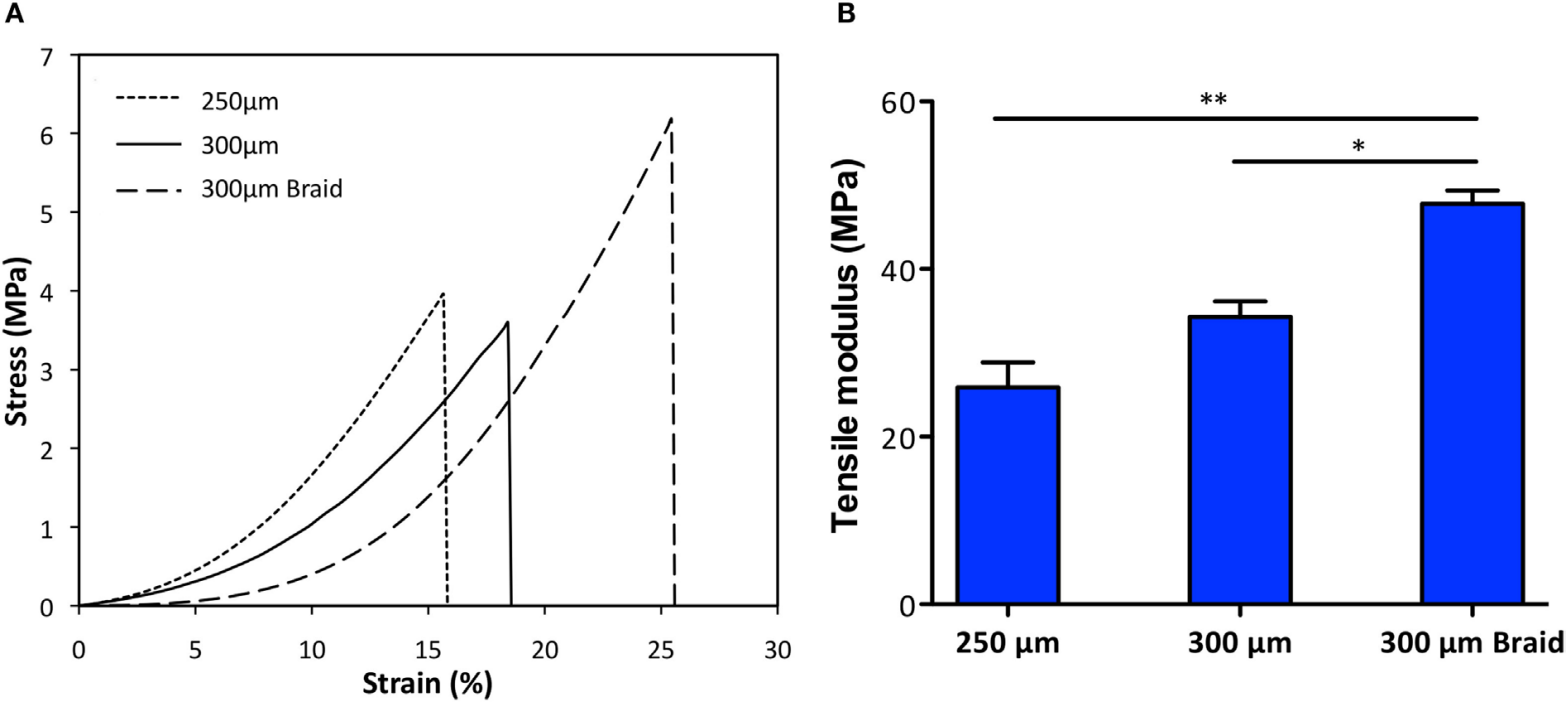
**Comparison of stress-strain curves (A) and tensile modulus (B) of the two most suitable membranes 250 μm and 300 μm thick and the braid-core obtained by interlacing the 300 μm thick membrane**. Samples had been previously hydrated. *p < 0.05, **p < 0.001.

The core component was obtained by a tape-casting process employed to distribute the CBE gel in a uniform layer, followed by an air-drying process to confer to the membrane an increased density, and to enhance the scaffold tensile modulus, preventing mechanical failure during *in vivo* implantation. The core component, with the final appearance of a braided membrane, achieved by enlacing three strips in wet condition (Figure 2A), is the main responsible for the composite scaffold mechanical properties in terms of strength and elasticity. With this approach, the stiffness in wet condition can be modulated as established from the mechanical evaluations (Figure 3).

#### Morphological Analysis

Scanning electron microscope analysis performed on air-dried CBE membranes shows a compact and regular surface made of a network of collagen fibers with different diameters homogeneously distributed and locally isotropically oriented. The surface presents only few deep grooves ranging from 1 to 5 μm suitable to allow cell adhesion and to support them during the migration along the scaffold (Figure 2B).

#### Enzymatic Degradation Test

The stability of the BDDGE cross-linked CBE-membrane was evaluated in terms of its degradation *in vitro* in presence of the enzyme collagenase. Membranes made of pure not cross-linked collagen were used as reference materials. The degradation profiles (Figure 2C), obtained by analyzing the same amount of samples with the same concentration of enzyme, clearly show the stabilizing effect given by the reticulation process, in fact, a complete degradation of the cross-linked sample was reached in 8 h while 3 h was sufficient for the complete degradation of the non-cross-linked membranes. In these experiments, higher concentration of collagenase has been used in comparison to the amount of enzyme found *in vivo* in order to accelerate the chemical digestion process, although the bacterial collagenase used is functionally different from the mammalian collagenases and this must be kept in mind.

#### Mechanical Properties

A preliminary screening was performed to select the most suitable CBE membranes (150–400 μm in thickness) in terms of stability, manageability, and elasticity: the 150-μm thick membrane resulted too thin and so extremely deformable, and the 400-μm membrane resulted too thick and so extremely stiff. Both the 250- and 300-μm thick membranes exhibit good stability and resistance, with a deformation degree of 4.1 and 3.2%, respectively, measured at the end of the applied stress cycle. The two most performing specimens (250 and 300 μm thick), shaped as a braid, were further investigated by evaluating the tensile modulus by DMA in simulated body conditions (PBS and 37°C) (Figure 3). Results show that the mechanical performances of the thicker membrane differed from the thinner one. The average tensile modulus of the thicker (34.3 ± 3.2 MPa) was higher than the thinner (25.9 ± 5.1 MPa) but lower than the braided samples (47.8 ± 2.7 MPa). These data confirmed that by interlacing the 300-μm thick strips in a braid, it is possible to reach higher mechanical performances than with the single strip. Moreover, the registered stress–strain curves highlight that all the specimens exhibit viscoelastic behavior typical of biological materials and due to the interaction of collagen with protein (like elastin) and water. This behavior can be ascribed to the mainly isotropic fibers arrangement, as evidenced from SEM images (Figure 2B), that in wet conditions and under the effect of unidirectional strain leans toward a gradual alignment. *In vivo*, fiber alignment under the effect of bio-mimicking strain represents a relevant cue that can stimulate proper cell migration and scaffold integration (Curtis et al., 2005).

### Characterization of the Shell Component

**Figure 4:**
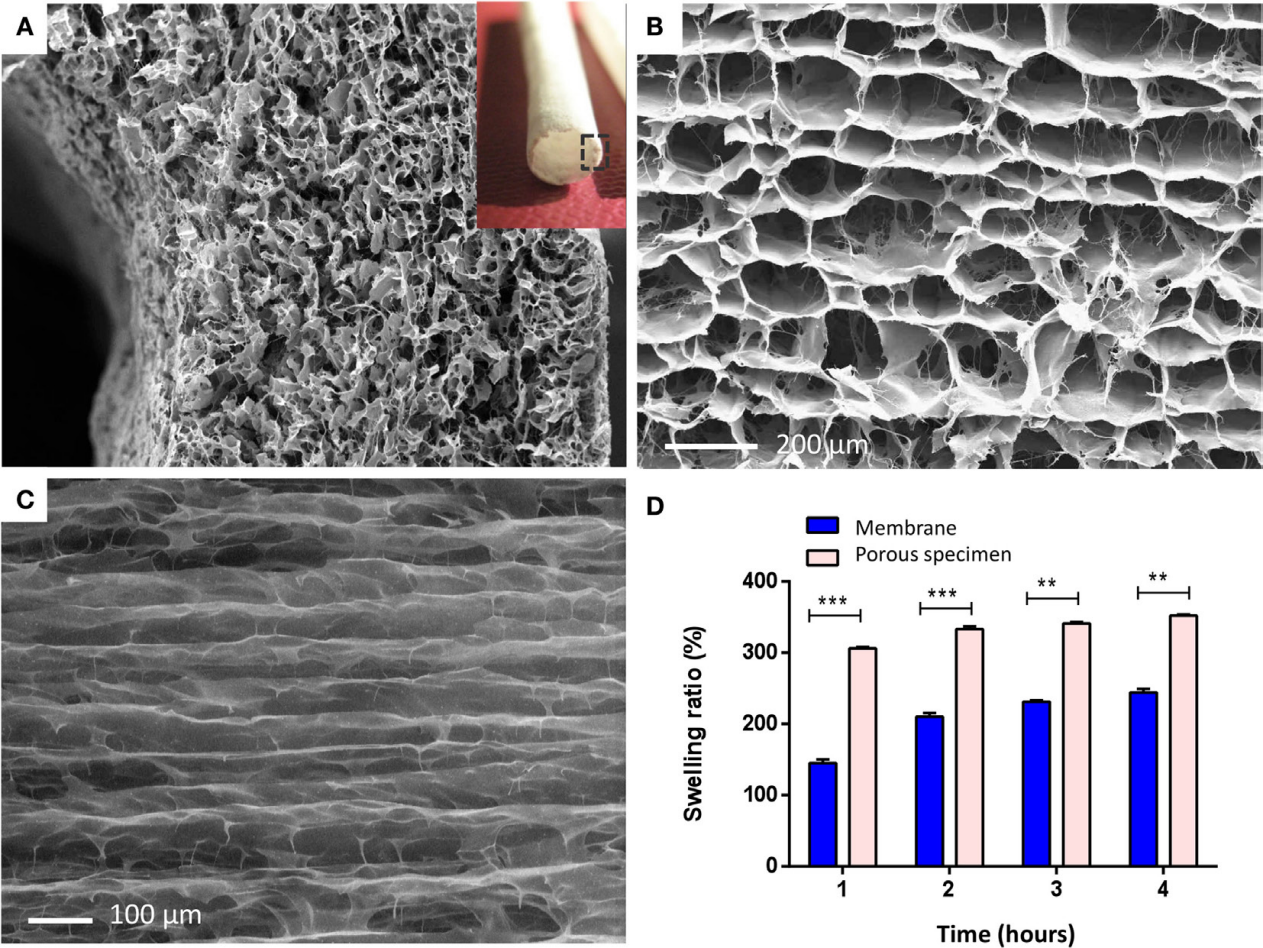
**(A)** Micro and macro (insert) structure of the shell scaffold. Image shows SEM scan performed on a transversal section of the tubular structure, showing the peculiar porous architecture of the tube wall, characterized by a sharp axially oriented pore distribution. **(B)** Axial view of the shell pore microstructure, endowed with an anisotropic axial porosity. **(C)** Longitudinal view showing preferential pore orientation along the axis of the tube. **(D)** Swelling behavior of the porous shell specimen in PBS solution, compared with the CBE membrane (300 μm thick) ***p ≤ 0.0001; **p ≤ 0.001.

The second component of the scaffold, namely the shell, was shaped as an hollow cylinder suitable to surround the core component and characterized from an high, longitudinal, and interconnected porosity able to adsorb physiological fluids and support and guide the cell migration all along the scaffold when implanted. It was fabricated by means of an uniaxial-freezing technique coupled with a standard freeze-drying process applied to the CBE gel and aimed to manufacture a hollow cylindrical device (i.e., a tube) endowed with an anisotropic porous architecture (i.e., highly oriented pore channels) (Figures 1 and 4A–C). Freeze-drying is a well-established process for the fabrication of porous structures where the sublimation step results in the complete removal of the frozen solvent from the final scaffold structures (O’Brien et al., 2004, 2005). Also, it is worth noting that by varying the freezing protocol (i.e., freezing temperature, freezing rate, direction of temperature gradient, etc.) together with collagen concentration of the CBE gel, the abovementioned uniaxial-freezing technique, combined with freeze-drying, allows for the production of porous construct displaying tailored porous microstructure or “micropatterning,” in terms of both pore size and orientation. In this work, the accurate modulation of the freezing and freeze-drying parameters, allowed us to produce hollow structures with a sharp micro-patterned porosity, characterized by longitudinally (i.e., along the tube axis) oriented pore channels within the tube wall. Such a porous structure could potentially host tendon cells, thanks to its open-cell pore structure and surface/bulk chemistry, but also to affect their infiltration and migration pattern, by means of its contact guidance properties, thus further supporting and accelerating tissue regeneration.

#### Morphological Analysis

Scanning electron microscope analysis performed on the shell scaffold (Figures 4A–C) shows the typical porous structure of a freeze-dried specimen, obtained by means of uniaxial freezing, whose network is characterized by a narrow pore size distribution, and a sharp pore micropatterning, with preferential orientation along the axis of the tube. Because of the features of this component, high porosity and mean pore-channel diameter of 150 µm, required to support tenocytes (50–100 µm in dimension) colonization and migration, it is characterized from poor mechanical properties. This property is not evaluated in this work because of poor significance for the role assigned to this component.

#### Swelling Behavior of Both Components

The swelling behavior of the shell scaffold was analyzed in comparison to the CBE-based membrane (base element of core component). As expected, the shell presents a swelling behavior characterized by higher volume ratio and absorption speed (Figure 4D), with respect to the compact membrane constituting the core component. This is due to its higher porosity, further showing that the unidirectional freezing process is a suitable technique for the development of tailored porous structures, potentially able to absorb physiological fluids when *in vivo* and to support and guide tenocytes during the regenerative process *in vivo*.

### *In Vitro* Cell Morphology Analysis on CBE Core and Shell Modules

**Figure 5:**
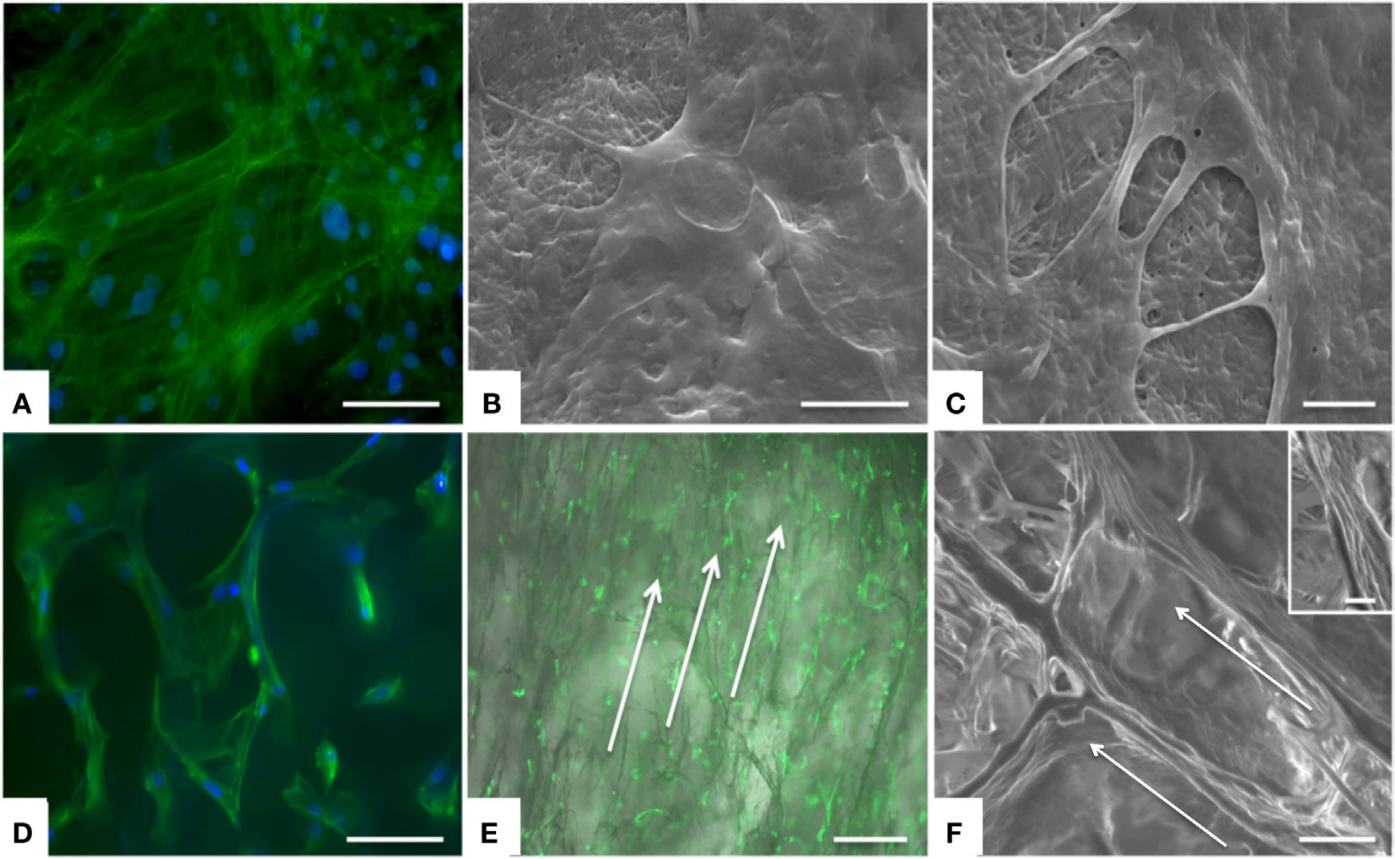
**Analysis of cell morphology upon culture on the device, assessed by actin staining and SEM**. MSCs exhibited good morphology and distribution on both CBE membranes **(A–C)** and CBE porous scaffold **(D–F)**. On CBE porous scaffolds, cells are aligned following the porosity of the sample. Arrows indicate cell alignment along the scaffold **(E,F)**. **(A,D)** Actin staining (green) and DAPI (nuclei, blue) at day 3, scale bar: 100 μm; **(B,C,F)** SEM at day 3, scale bars: 20 μm, 5 μm, 50 μm, respectively; insert of **(F)**: a detail of cell grown on CBE porous scaffold; **(E)** Actin staining with brightfield background at day 1, scale bar: 500 μm.

Morphological analysis by specific staining of actin filaments enabled the examination of MSCs properly distributed on both CBE membranes and CBE porous scaffolds (Figures 5A,D). In detail, for swelled CBE membranes, MSCs showed their characteristic cytoskeleton morphology (Figure 5A) and fine cellular morphology by SEM confirmed that cells were well spread on the CBE membrane surface, indicating biocompatibility and bio-mimicry of the material (Figures 5B,C). With regard to three-dimensional CBE porous samples, cells were seen in tight relationship with the scaffold microporous topology, well growing on scaffold fibers (Figure 5D). Indeed, due to the high porosity of the scaffold, it was easily colonized by cells already at day 1 (Figure 5E); also, a clear alignment of MSCs along the scaffold porosity pattern was evidenced by both actin staining (Figure 5E, arrows) and SEM (Figure 5F, arrows). The maintenance of the characteristic MSCs morphology shown *in vitro* for both the core and the shell materials may be considered as an indicator of biocompatibility (Kumari et al., 2001; Matsuoka et al., 2013). Moreover, the shell scaffold porous architecture deeply influenced either cell infiltration or alignment within the scaffold itself. Therefore, due to its chemical and morphological features and consequently of its high cell permeability, the shell module may behave like a guide and a reservoir for cells, giving a relevant boost to the regenerative process, by supporting *in situ* scaffold integration and tendon regeneration.

### *In Vivo* Implantation of the CBE Core Module in a Rat Tendon Defect

**Figure 6:**
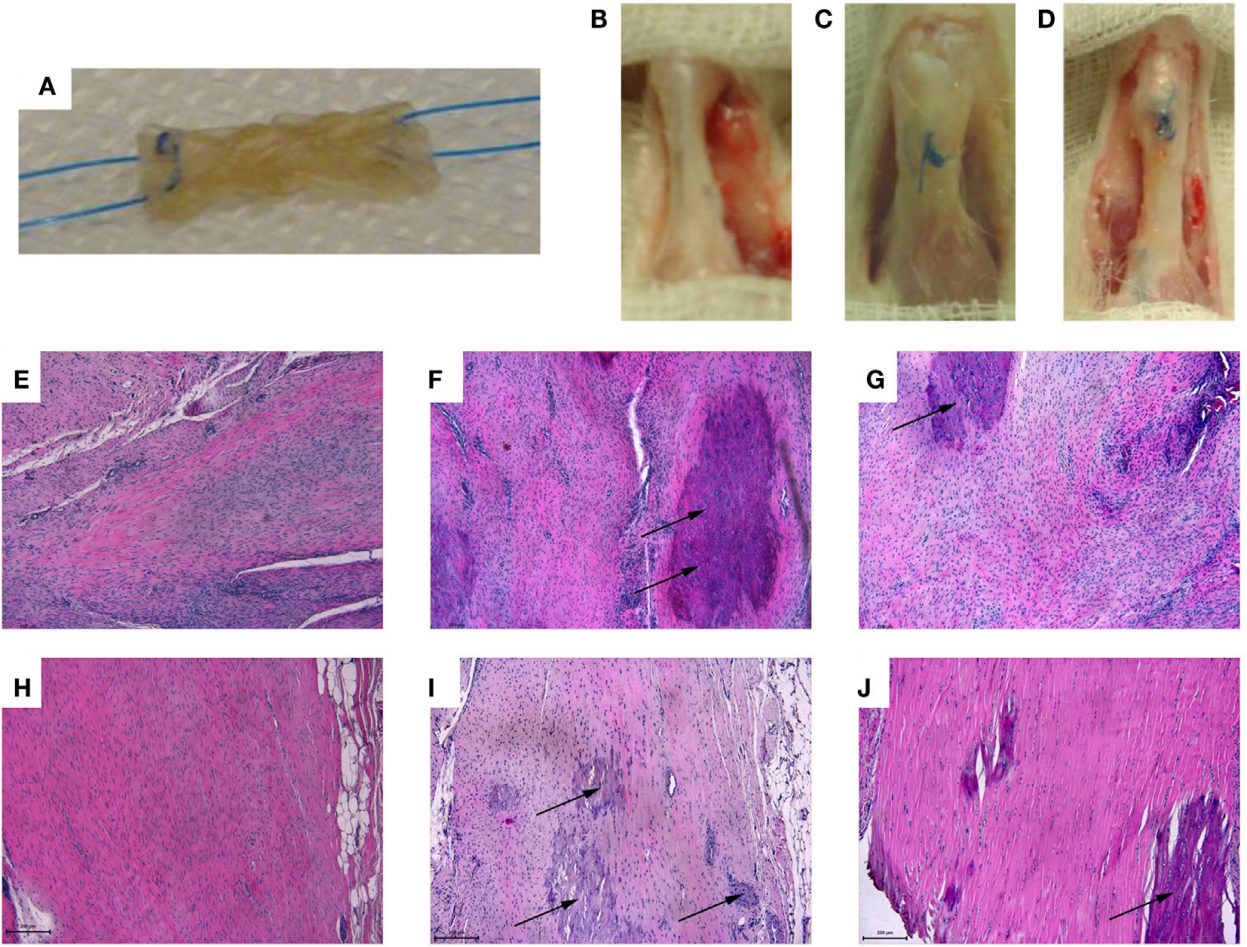
**(A)** Core module before *in vivo* implantation: the device was appropriately sized to fit the created tendon gap, and anchored through a 6–0 suture with a Kessler suture technique to ensure stable fixation. **(B–D)** 16 weeks explants: **(B)** sham: the tendon was isolated from the surrounding tissues but no tendon lesion was created; **(C)** control: 2/3 of the tendon was removed and a terminal-terminal suture was done to maintain the stumps at a correct distance; **(D)** treated area: the gap area had been filled by the scaffold. **(E–J)** Longitudinal sections of the rat right Achilles tendons stained by Hematoxylin and Eosin from: sham **(E,H)**, control **(F,I)**, and treated **(G,J)** groups at 8 weeks **(E–G)** and 16 weeks **(H–J)** after surgery (magnification, 10×, scale bar = 200 μm). A loss of organization of the collagen bundles with an increased number of connective tissue cells and tenocytes at the injury site was found in all groups. **(E,H)** Degenerated tendons with irregular collagen fibril arrangement and evident hypercellularity. **(F,I)** Highly degenerated tendon with some chondroid cells (arrows), yet absence of inflammatory cells. **(G)** Slightly pathological tendinous tissue with initial matrix disorganization and evident hypercellularity; note the presence of chondroid cells (arrows). **(J)** Uniform appearance of non-compressed and well aligned collagen fibrils with interspersed tenocytes aligned parallel to the fibrils; note the presence of chondroid cells inside the tendon (arrows) and the great number of fibroblasts in the tendon’s external region.

The developed tendon prototype was object of short-term *in vivo* implantation in order to achieve preliminary data on the performance of the implant in a preclinical model. A rat model was used in order to receive indications regarding safety, feasibility, and clinical potential of this collagen-based construct. Only the core component (mechanically competent) of the scaffold was implanted in order to reduce the complexity grade of the suture step, since the rat model would not allow a double layer suture for the entire core-shell graft (Figure 6A).

All the animals well tolerated the surgery without clinical signs of necrosis or tissue infection, except for two rats of the control group that died 1 day after surgery due to anesthesiologic complications and were replaced. The gross appearance of the repair site at time of dissection was different between groups, and noteworthy the tendons of treated group were more elongated than those of control and sham groups. No macroscopic signs of necrosis or tendon degeneration were observed (Figures 6B,D). The histological observations of operated Achilles tendons described here were consistently found in all samples within each group (Figures 6E–J).

**Figure 7:**
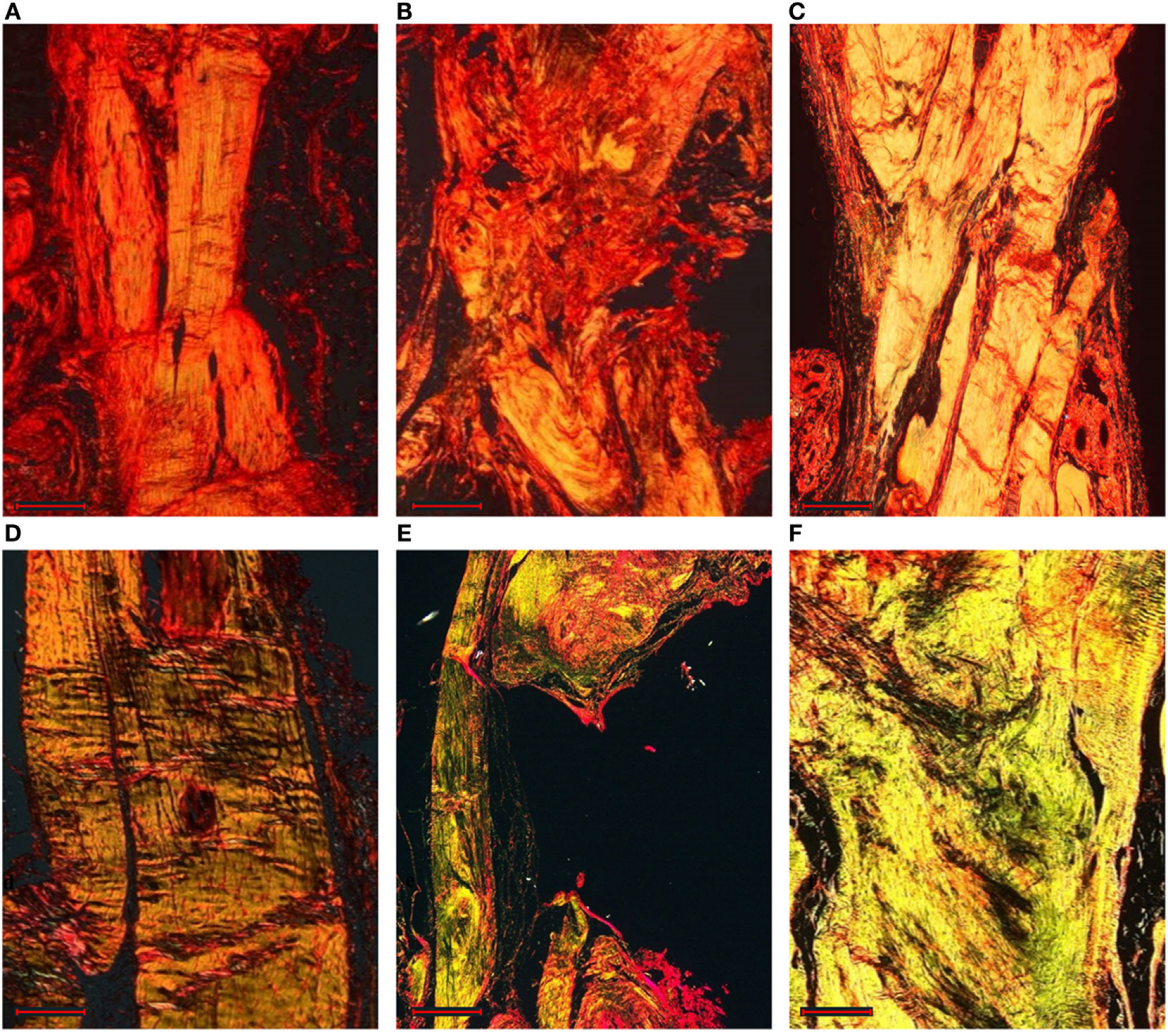
**Longitudinal sections of the rat right Achilles tendons stained by Picro-Sirius red staining: sham (A,D), control (B,E), and treated (C,F) groups at 8 weeks (A–C) and 16 weeks (D–F) after surgery (magnification, 4×, scale bar = 500 μm)**. The thicker and mature collagen fibers appear red-orange (type I collagen); the thinner collagen fibers appear pale green (type III collagen).

The normal parallel alignment of long wavy collagen fibers, where fibroblast rows are situated, was observed in all healthy left Achilles tendons (data not shown). The membrane that covers the tendon, as well as the collagen fibers of the epitenon, was thin, and no inflammatory infiltrate or other morphological changes were seen. Except for some cases of control group, where no tissue was found between tendon gaps, microscopic examination identified pronounced differences between tendons of control group and the other operated groups. In general, the pathological region was distinct from the normal tendon with both matrix and cellular changes. Instead of clearly defined, parallel and slightly wavy collagen bundles, loss of the longitudinal alignment of collagen fibers and loss of the clear demarcation between adjacent collagen bundles were found at different levels in all observed right tendons. Eight weeks after surgery, a particular appearance of regrown and healed tendons inside the scaffold was observed, with a distribution of tenocytes in bundles with still disorganized and separated fibers along the spiral structure of the scaffold. Sixteen weeks after surgery, in the sham group, macroscopic evaluation shows a normal tendon structure (Figure 6B), in the control group, macroscopic evaluation shows a fibrous appearing scar tissue at the suture level (Figure 6C), in the treated group, the gap area had been filled by the scaffold, new formed tissue can be observed with a continuous but elongated regenerated tendon between the still detectable stumps sutures (Figure 6D). From the histological analysis (Figures 6E–J and 7), the distribution of collagen fibers and tenocytes was similar to that of sham group. The treated and control tendons were characterized by the presence of numerous multiple cellular changes, such as hypercellularity and increased in roundness.

**Figure 8:**
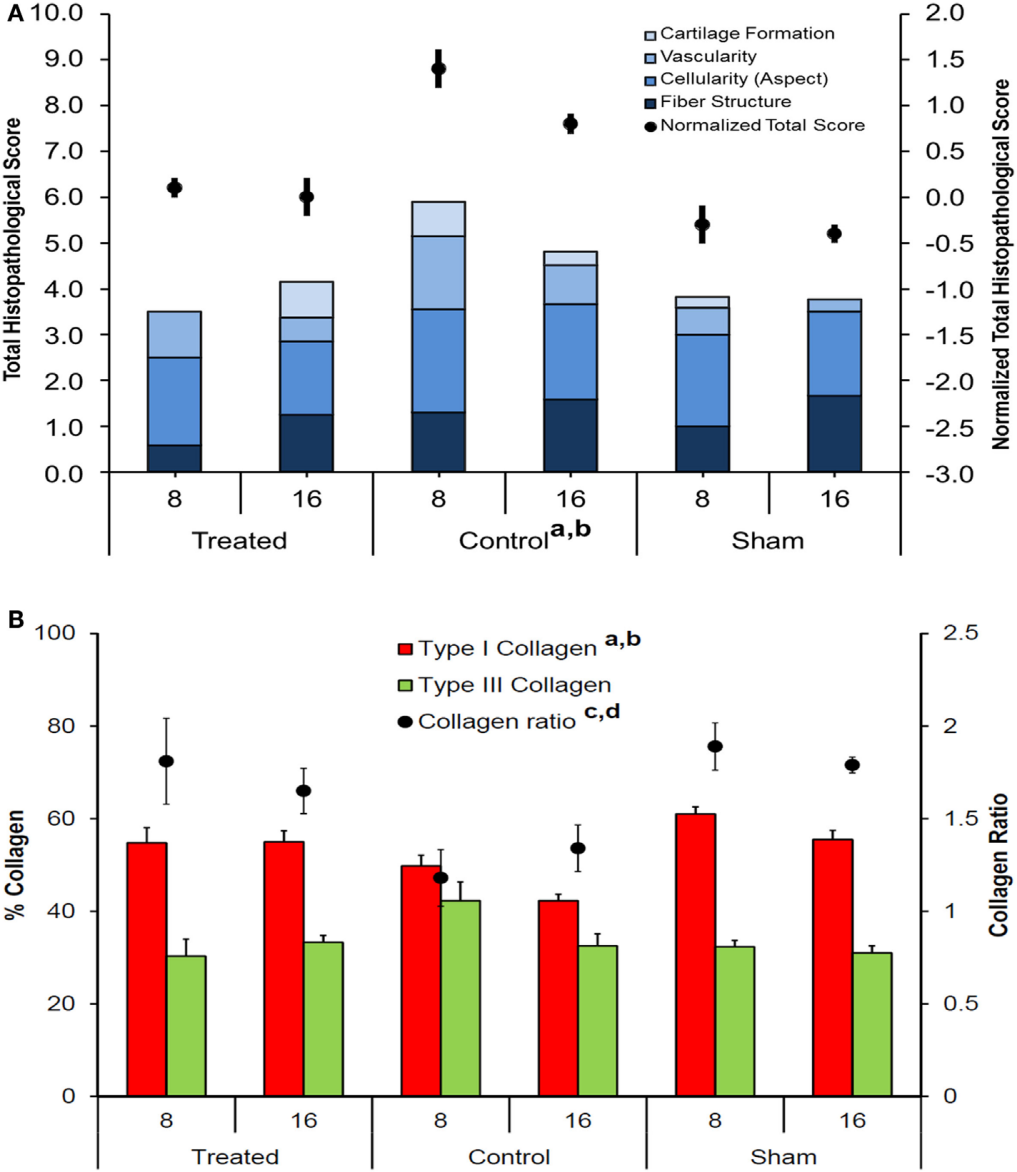
**(A)** Stacked histograms of the semiquantitative histomorphometric score for treated, control, and sham groups, including cartilage formation, vascularity, cell aspect, and fiber structure scores (Mean, *n* = 5). Dots represent the normalized total score for healthy tendon score for each group (mean ± SEM, *n* = 5); adjusted Bonferroni *t* test: treated versus control group (^a^*p* < 0.005); control versus sham group (^b^*p* < 0.0005); **(B)** Histograms of Type I and Type III collagen content, expressed in percentage, for treated, control, and sham groups (mean ± SEM, *n* = 5). Dots represent the Collagen Ratio (Type I/Type III) for each group (mean ± SEM, *n* = 5); adjusted Bonferroni *t* test: treated versus control group (^a^*p* < 0.05) and control versus sham group (^b^*p* < 0.005).

Independently of the group, most of the tendons showed areas of hyalinization due to the presence of fibro cartilaginous metaplasia. This phenomenon could be related mainly to surgical procedures. Semiquantitative histomorphometric results are reported in Figure 8A. GLM analysis highlighted significant effect for “group” (*F* = 19.40, *p* < 0.0005) and no effect for “experimental time” on “Normalized Total Score.” No interaction of the two effects was found on “Normalized Total Score.” Independently of experimental times, “Normalized Total Score” of control group was significantly higher than that of treated (1.0, *p* < 0.005) and sham (1.4, *p* < 0.0005) groups. Regarding “Collagen Ratio” results, GLM analysis showed a significant effect for “group” (7.44, *p* < 0.005) and independently of experimental times, the lowest values were found in control group in comparison to treated (−0.51, *p* < 0.05) and sham (−0.57, *p* < 0.005) groups. (Figure 8B). Overall, these results indicate a positive effect on tendon regeneration given by the implant. However, despite the good histological results, the properties of the implanted scaffold were still insufficient to meet the *in vivo* requirements, as demonstrated by the elongation documented in the explants of the treated animals; however, this effect should be ascribed to the fact that rats were left free to move and load the operated area immediately after the operation, thus without any graft protection throughout the experimental time.

Further improvement of this scaffold is required before a clinical application, mainly to improve the mechanical requirements before the biological reconstruction of a tissue with the properties of a healthy tendon. A possible immediate use of this prototype is its application as augmentation biomaterial in surgical procedures where mechanical resistance is guaranteed by tendon remnant or hardware positioned to protect the implant during the maturation phase. A potential human application could benefit from a period without weight bearing and from the protection of a brace in the first healing phases, thus allowing not to jeopardize the tendon and to favor its maturation without excessive loading. Limitations of our study are the lack of a mechanical evaluation of the explanted tendons and the absence of a control group where the implants were protected during the first healing phase. However, this *in vivo* study allowed us to document the safety and feasibility of this scaffold with no adverse tissue reactions and to underline its potential in favoring tendon regeneration. Based on the achieved preliminary results, the tendon prototype in its complex design (including both modules) will be involved in a long-term *in vivo* study following a large-size animal protocol. In these conditions, the functional support of the shell component working as cells guide and reservoir will be of crucial importance to balance the low cell permeability of the core component ascribable to its poor porous surface made of well-compacted collagen fibers, thus possibly leading to a better quality and faster tissue regeneration.

## Conclusion

A new collagen-based 3D tendon prototype, endowed with biomimetic chemical–physical properties, was designed and developed – thanks to fabrication techniques that are able to generate specific geometries and pore structures in compliance with the target tissue.

Collagen-BDDGE-elastin suspension was developed and processed by a tape-casting and uniaxial-freezing methods to achieve bioresorbable scaffolds with the feature of membrane endowed with suitable mechanical performances and chemical stability, suitable to lead the adhesion, migration and proliferation of cells before its complete biodegradation and of 3D porous scaffolds characterized by longitudinally oriented pore channels thought as a guidance matrix, suitable to host, support, and guide tenocyte toward the gap of the damage tissue.

By combining both components, the prototypes with a “core–shell” structure have been successfully obtained and characterized in view of their potential application as tendon scaffolds for tissue regeneration.

The preliminary short-term *in vivo* study performed following a small-size animal protocol on the mechanically competent component, documented the safety, and the good performance of the core module of the device. Concerning the shell, it is expected that cells *in vivo* will be stimulated to infiltrate and colonize its highly porous and isotropic structure, guided by the preferential micropatterning and contact cues imparted from the high biomimetic collagenic matrix, therefore facilitating tendon regeneration. The synergic action of both modules, the core for its essential mechanical support and the shell acting as tendon guidance matrix, offers a promising solution for tendon regeneration with good results in terms of cell adhesion and tissue quality and functioning.

On the basis of the preliminary results showed in this work, the scale-up of the new core-shell CBE prototype toward larger scaffold for the assessment of its regenerative potential in more clinically reflective animal models, can be considered a matter worthy of investigation.

## Author Contributions

MS performed the research, analyzed the data, and wrote the paper. GF designed the research study concerning the *in vivo* test, performed the research, and wrote the paper. EK designed the research study concerning the *in vivo* test and performed the research. SP performed the research concerning the *in vitro* test and analyzed the data. MMo performed the research concerning the *in vitro* test and analyzed the data. MI wrote the paper. ES performed the mechanical evaluations. SS analyzed the data and wrote the paper. CC revised and restructured the manuscript. GG analyzed the data and wrote the paper section concerning the *in vivo* evaluation. FV performed the research concerning the *in vivo* test and analyzed the data. MF performed the research concerning the *in vivo* test and analyzed the data. LS designed the research study concerning the device development. AS designed the research study concerning the device development. MMa designed the research study concerning the *in vivo* test. AT designed the research study concerning the device development.

## Conflict of Interest Statement

The authors declare that the research was conducted in the absence of any commercial or financial relationships that could be construed as a potential conflict of interest.
